# Influence of migrant background on patient preference and expectations in breast and gynecological malignancies (NOGGO-expression V study): results of a prospective multicentre study in 606 patients in Germany

**DOI:** 10.1186/s12885-021-08731-6

**Published:** 2021-09-12

**Authors:** D. Dimitrova, B. Naghavi, R. Richter, S. Nasser, R. Chekerov, E. I. Braicu, M. David, J. Blohmer, G. Inci, U. Torsten, G. Oskay-Özcelik, I. Blau, N. Fersis, A. Holzgreve, E. Keil, M. Keller, U. Keilholz, J. Sehouli

**Affiliations:** 1 Department of Gynecology with Center of Oncological Surgery, Charité University Medicine, Campus Virchow-Klinikum, Berlin, Augustenburger Platz 1, 13353 Berlin, Germany; 2 Charité Comprehensive Cancer Center, Charité University Medicine, Berlin, Germany; 3 Department of Gynecology and Breast Care Center, Charité University Medicine, Charité Campus Mitte, Berlin, Germany; 4 Department of Gynecology, Vivantes Klinikum Neukölln Berlin, Berlin, Germany; 5 Gynecological Oncology Medical Practice Berlin Spandau, Berlin, Germany; 6 Medical Care Center Evangelisches Waldkrankenhaus am Standort Pankow, Berlin, Germany; 7 Helios Klinikum Duisburg, Duisburg, Germany; 8 Vivantes Netzwerk für Gesundheit GmbH, Berlin, Germany; 9 Klinik Oranienburg, Oberhavel Kliniken GmbH, Oranienburg, Germany; 10 North-Eastern-German Society of Gynecological Oncology, Oranienburg, Germany

**Keywords:** Migrants, Survey, Doctor-patient relationship, Patient preference, Therapy expectations

## Abstract

**Background:**

An effective cross-cultural doctor-patient communication is vital for health literacy and patient compliance. Building a good relationship with medical staff is also relevant for the treatment decision-making process for cancer patients. Studies about the role of a specific migrant background regarding patient preferences and expectations are lacking. We therefore conducted a multicentre prospective survey to explore the needs and preferences of patients with a migrant background (PMB) suffering from gynecological malignancies and breast cancer to evaluate the quality of doctor-patient communication and cancer management compared to non-migrants (NM).

**Methods:**

This multicentre survey recruited patients with primary or recurrence of breast, ovarian, peritoneal, or fallopian tube cancer. The patients either filled out a paper form, participated via an online survey, or were interviewed by trained staff. A 58-item questionnaire was primarily developed in German and then translated into three different languages to reach non-German-speaking patients.

**Results:**

A total of 606 patients were included in the study: 54.1% (328) were interviewed directly, 9.1% (55) participated via an online survey, and 36.8% (223) used the paper print version. More than one quarter, 27.4% (166) of the participants, had a migrant background. The majority of migrants and NM were highly satisfied with the communication with their doctors.

First-generation migrants (FGM) and patients with breast cancer were less often informed about participation in clinical trials (*p* < 0.05) and 24.5% of them suggested the help of an interpreter to improve the medical consultation. Second and third-generation migrants (SGM and TGM) experienced more fatigue and nausea than expected.

**Conclusions:**

Our results allow the hypothesis that training medical staff in intercultural competence and using disease-related patient information in different languages can improve best supportive care management and quality of life in cancer patients with migrant status.

**Supplementary Information:**

The online version contains supplementary material available at 10.1186/s12885-021-08731-6.

## Background

Migration is a global phenomenon with 244 million international migrants worldwide [1]. In Germany, one quarter (25.5%) or 20.8 million German citizens have a migrant background [2]. The group of people with a migrant background is heterogenic, including those with their migration history or those who are offspring of migrants born in the host country, where at least one parent has another background. Because migrants are not often considered for medical trials, only a few data focuses on patients with a migration background (PMB) [3].

According to previous studies, people with a migration background have specific health-related risk factors and health-related behavior [4]. Some specific issues faced by PMB are the need for acculturation in the host country, language barriers, and differences in cultural values. These factors may influence the treatment decision-making process for doctors and cancer patients. Establishing an adequate cross-cultural doctor-patient relationship may be crucial to overcome these barriers and facilitate the exchange of information and include patients in the decision-making process [5].

There are lacking data for PMB with gynecological malignancies and with breast cancer. Therefore, the present study was initiated. The objectives of this pilot study are to gather information about the preferences of patients regarding medical treatment and communication with their doctors. The main outcomes of the study are to measure the patient satisfaction with different aspects of the medical consultation, to evaluate patient satisfaction with their involvement in cancer treatment and participation in clinical studies and to assess patient demographics. The secondary goals are to assess patient satisfaction with the therapy results, to evaluate the therapy side effects and gather their perspectives of improving health care for migrants and disease etiology. The survey focuses on different migrant generation patients with gynecological malignancies and with breast cancer. The study population included both patients with and without a migrant background. This study aimed to describe patient expectations and preferences and develop future interprofessional and interdisciplinary strategies to improve patient management of women with and without a migrant background. For this study, we use “migrants” when we refer to people with a migrant background. A particular focus of this study is to evaluate the participation of PMB in oncology clinical trials.

## Methods

The study was carried out in two steps between August 2014 and August 2017 at 19 gynecologic and oncology departments (*n* = 11) and practices (*n* = 8) in Germany.

Patient participation was via an online survey and a paper print version. For the Berlin region, additional personal interviews were carried out in four languages (German, Russian, Turkish, Arabic) by trained staff who did not have any involvement in patient management. After a run-in phase with 15 centers in Berlin (2014–2015), the study was extended by including 4 additional departments from different federal states in Germany (2016–2017).

All gynecologic oncology departments of maximum and main care and practices with a focus on gynecologic oncology in Berlin were invited to participate in this study. The criteria to choose the clinic were if they have reported “gynecological oncology” as a treatment focus. Seven out of 15 eligible departments declared their interest and participation. Additionally, we invited 21 practices with focus on “gynecological oncology” in Berlin to participate and 8 responded and participated in the study.

Additional to the 15 centers in Berlin, 4 gynecological departments of maximum and main care clinics from 4 different federal states of Germany with the largest number of inhabitants asked to participate (Bayern, Nordrhein-Westfalen, Niedersachsen, Baden-Württemberg).

The Charité Ethics Committee (EA1/059/14) and the Charité Office for Protection of Data Privacy approved the study protocol to include patients via an online survey, paper printed questionnaire, and interviews.

For all the different data collection possibilities, an approved patient information sheet was provided before participation. According to the ethics protocol, patient signatures were not required in order to preserve anonymity on the informed consent form. The sheet expressly declared that by answering the questions, the responder agreed to participate in this survey voluntarily, and the answers would be further analyzed and published. Furthermore, it was stated that participation was optional and that non-participation would have no consequences in further treatment.

In the internet survey, the same algorithm was used and the information sheet was placed electronically. The participants were automatically redirected to a patient information sheet, had to read it and confirm their agreement to proceed with the questionnaire.

The survey recruited patients with primary or recurrence of breast, ovarian, peritoneal, or fallopian tube cancer. Based on the experience of previously published large multicentre surveys [6, 7], an interdisciplinary and interprofessional team developed a 58-item questionnaire. It was translated into three different languages: Russian, Arabic, and Turkish due to the high prevalence of these languages in Germany. Bilingual medical staff validated the translation of the questionnaire.

The survey was divided into three sections: the first one included questions for assessing the migrant background and demographics, the second was related to the course of disease including questions about the type of cancer, the type of cancer therapy, and the current therapy situation. The third section included questions about patient preference, expectations, and evaluation of their level of satisfaction in medical communication, involvement in health services and patient preferences on therapy and doctor-patient communication. The objective of the survey was to gather information, not to implement a specific questionnaire. Therefore, no statistical validation tests were applied.

The paper printed form was generally provided during a consultation by the physician in one of the patient’s preferred languages; German, Arabic, Russian or Turkish.

The interviews took place in the following four maximum care hospitals in Berlin: Charité Campus Virchow Klinikum Berlin, Charité Campus Mitte (University of Berlin), Vivantes Klinikum Neukölln, and Vivantes Klinikum am Urban.

The bilingual medical staff was trained for the survey. They conducted the interviews in the patient’s preferred language (native) using the 58-item questionnaire as a template during a hospital stay, during outpatient appointments for a follow-up examination, or during chemotherapy in an outpatient setting. The interviewers were explicitly trained and were supervised by professional staff before the start of the study. As a result, migrants with insufficient German language skills could also be included. In this case, the participants were interviewed in Russian, Arabic, or Turkish. Only patients who had already received cancer therapy such as surgery, chemotherapy or were currently undergoing cancer treatment were considered for the interviews. None of the interviewers were involved in the clinical management of the patients to minimize additional bias. Newly diagnosed women or women who had not started any therapy at that time were excluded from the interviews to achieve comparable results.

The online survey was available in 4 languages: German, Russian, Arabic, and Turkish. It was accessible via the study webpage to all the patients with a diagnosis of primary or recurrence of breast, ovarian, peritoneal, or fallopian tube cancer during the whole study period.

### Statistics

SPSS Version 25 was used for the statistical analysis. The focus of all analyses was descriptive and primarily performed for comparing the group of NM to FGM and SGM/TGM. For the definition of migrant background, the birthplace of the patient and both parents, the preferred language of communication, the level of spoken German, and the residency time in Germany were assessed [8]. If the patient and both parents were born in another country, the patient was considered to be a FGM. If the patient was born in Germany, but one of the parents was not, the patient was considered to be a SGM. If the patient and both parents were born in Germany but the preferred language was not German, the patient was considered to be a TGM.

The Chi-square-test was used for categorical variables to distinguish the differences between the NM and FGM and SGM/TGM. The non-parametric Kruskal Wallis Test was used to explore the differences in patient satisfaction in medical consultation, health care service and therapy results. The normal distribution of the variables was proved with the Kolmogorov-Smirnov-Test. A 10-point Likert scale was used for evaluating patient satisfaction regarding medical consultation; 1 expressing the lowest and 10 the highest level of satisfaction. A similar 10-point-scale was used to evaluate patient expectations with respect to the treatment results, where 1 represented “worse than expected” and 10 “better than expected.”

A ROC curve was used for defining the best cut-off age for the study population. A multivariate binary regression model was employed to test if non-participation in a clinical study was influenced by the patient’s migrant background, the level of German language skills, or their age. We performed a subgroup analysis for the type of cancer; breast cancer versus gynecological cancer from the mentioned migrant generations.

## Results

### Demographic characteristics of the study population

**Fig. 1:**
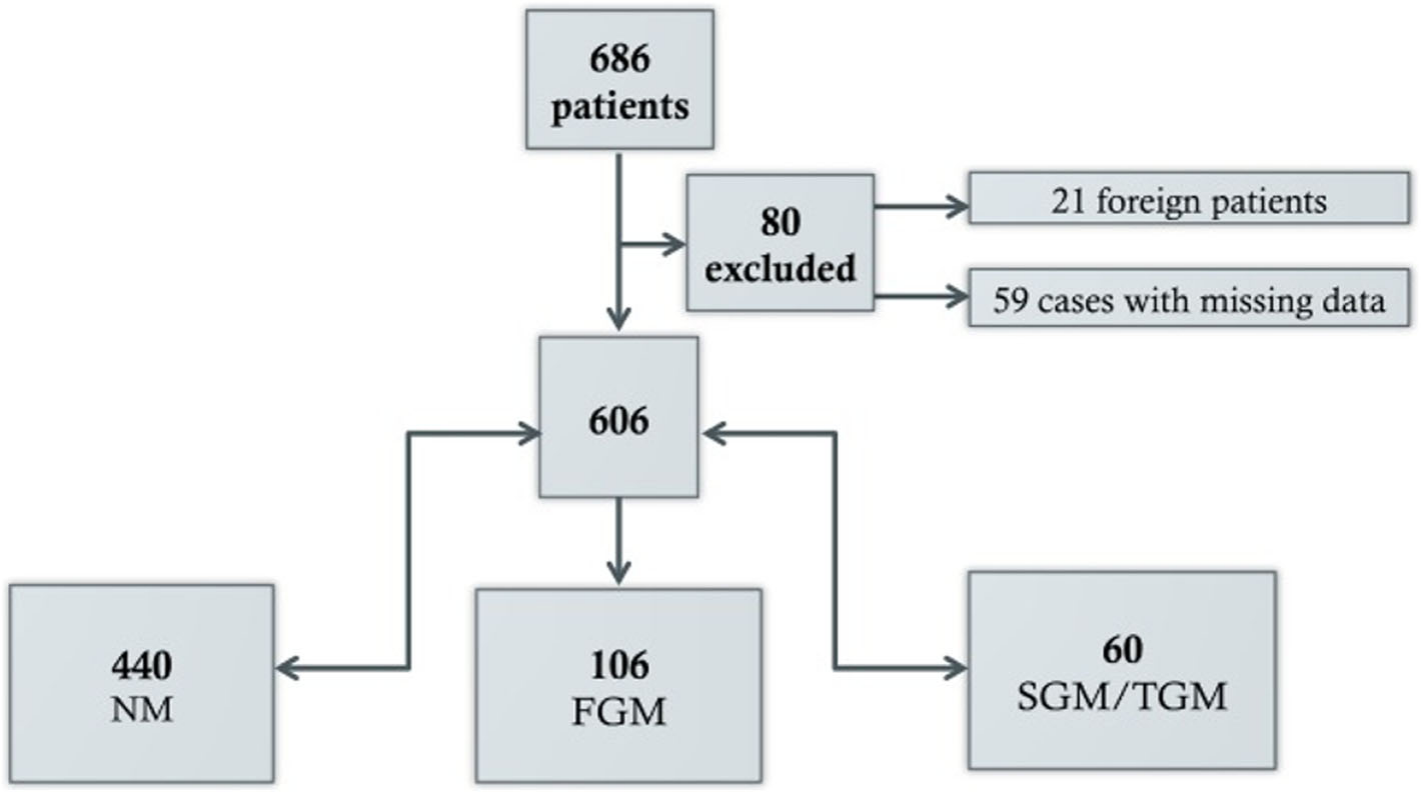
Flowchart for recruited patients. NM: Non-migrants, FGM: first-generation migrants, SGM: second-generation migrants, TGM: third-generation migrants

During the study period, 686 patients participated in the survey. Patients with missing data (*n* = 59) and who were not permanently living in Germany (*n* = 21) were excluded (see Fig. 1). Using our pre-defined criteria, 106 (17.5%) of the participants were classified as FGM, 57 (9.4%) as SGM and 3 (0.5%) as TGM. The last two groups were considered as one group SGM/TCM for further statistical analyses.

According to the preferred participation way, 328 (54%) patients were interviewed, and only a small number of the participants filled out the online questionnaire 55 (9.1%). The remaining 223 (36.8%) filled out the paper print form. There were no differences in the preferred way of participation between migrants and NM. The participants who chose the online form were significantly younger. Supplemental Table 1 describes the median age and migrant status by the chosen way of participation.

Concerning language preference, 54.6% of the migrants preferred to speak German, 21% Turkish, 16% Russian, and 4.2% other languages. Regarding migrant generations, only 16% of FGM preferred to speak German, 48.1% favored another language than German, and 35.8% were bilingual. In contrast, SGM/TGM, 83.1% preferred German, 11.9% were bilingual, and only 5.1% preferred another language.

Table 1 summarizes the demographic characteristics of the study population.

**Table 1:** Patient characteristics

	NM	FGM	SGM/TGM	*p*-value
**Demographics**				
**Number of patients (n)**	440	106	60	
Age (median)	59 (22–92)	52 (30–83)	59 (24–84)	< 0.05
Have children	322 (73.5%)	89 (84.0%)	38 (65.5%)	0.02
Married or living in a relationship	295 (68.1%)	76 (71.7%)	42 (71.2%)	0.72
Currently employed	162 (36.8%)	27 (26.0%)	18 (31.0%)	0.09
German as a preferred language	440 (100.0%)	17 (16%)	49 (83.1%)	< 0.05
German citizens	436 (99.3%)	37 (35.2%)	52 (86.7%)	< 0.05
More than 6 years in Germany	440 (100.0%)	86 (83.5%)	60 (100.0%)	< 0.05
Internet access	340 (77.6%)	83 (79.0%)	55 (91.7%)	0.04
**Level of education**				< 0.05
No education	1 (0.2%)	13 (12.5%)	0 (0%)	
Primary education	72 (16.5%)	21 (20.2%)	15 (25.4%)	
Secondary or tertiary education	208 (47.6%)	40 (38.5%)	22 (37.3%)	
University degree	156 (35.7%)	30 (28.8%)	22 (37.3%)	
**Disease**				
Ovarian, peritoneal or fallopian tube cancer	258 (58.6%)	44 (41.5%)	37 (61.7%)	0.004
Breast cancer	203 (46.3%)	63 (59.4%)	23 (39.0%)	0.02

We observed some differences between the FGM and NM in social status and German language skills. FGM were significantly younger (median age 52), were more often married, and had children. The percentage of employed was lowest among the FGM 26.0%, where 12.5% of them had no school education at all, 23.8% only had poor German language skills, and 3.8% could not speak any German. German was the preferred language for SGM/TGM, compared to FGM. Seventy-five percent of them rated their German language skills as very good and 23.3% as good. When comparing the mentioned generations in the breast cancer group and the gynecological cancer group, the following was observed. FGM patients were significantly younger (median age 49) than NM (median age 57), and SGM/TGM (median age 56) in the breast cancer group. In the gynecological cancer group, the patients tended to be older; FGM (median age 57), NM (median age 60), and SGM/TGM (median age 61). The cases with ovarian, peritoneal, or fallopian tube cancer were higher among SGM/TGM 61.7% (37 patients) in comparison to FGM 41.5% (44 patients), and NM 58.6% (258 patients). The majority of breast cancer patients were found in FGM 59.4% (63 patients) compared to NM 46.3% (203 patients) and SGM/TGM 39% (23 patients).

### Medical management and information needs

**Fig. 2:**
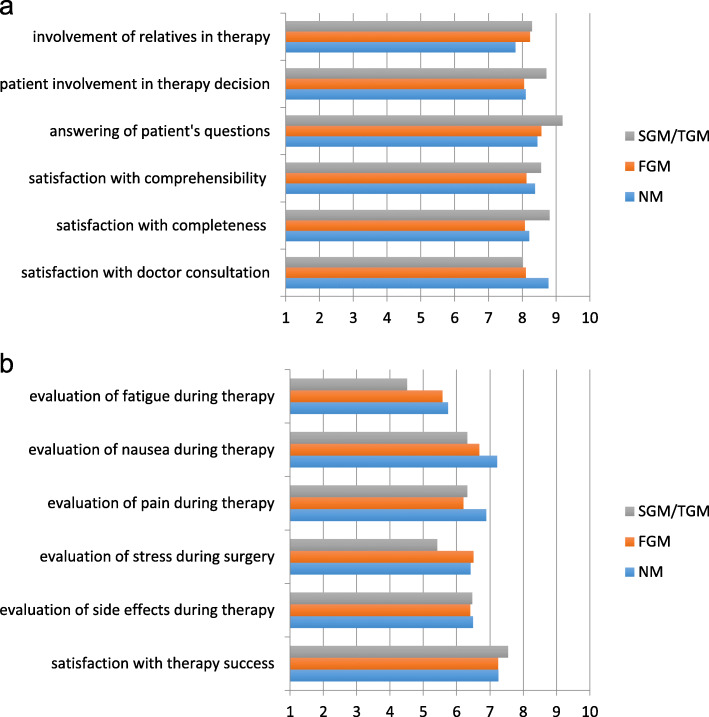
**a** Evaluation of the quality of doctor consultation, patient involvement in therapy decisions and involvement of patient’s relatives in the therapy on a 10-point scale, where 1 expresses the lowest level and 10 the highest. **2b** Evaluation of therapy expectations and therapy side effects on a 10-point scale, where 1 expresses “worse than expected” and 10 “better than expected”

There were no significant differences in patient satisfaction with global medical management. The majority of the patients were satisfied with the communication with their doctors. The mean score was 9.0 (on a scale of 1–10). There were no differences between NM and PMB when evaluating doctor-patient communication and patient involvement in the therapy (refer to Fig. 2a). Almost 25% of the FGM suggested the presence of a professional interpreter during the medical consultation to improve communication with people of various migrant backgrounds (*p* < 0.05) (refer to Table 2). Intercultural training for the medical staff to improve the care of migrants was suggested by 22.6% of them, and the employment of more doctors with migrant backgrounds was encouraged by another 35%.

**Table 2:** Proposed measures for improving cancer care

	NM	FGM	SGM/TGM	*p*-value
Shorter therapy	65 (14.8%)	22 (20.8%)	11 (18.3%)	0.29
Better cooperation between physicians	112 (25.5%)	26 (24.5%)	21 (35.0%)	0.26
More consultation time	134 (30.5%)	40 (37.7%)	27 (45.0%)	0.04
Therapies without alopecia	126 (28.6%)	29 (27.4%)	18 (30.0%)	0.93
More measures for pain prevention	41 (9.3%)	17 (16.0%)	10 (16.7%)	0.05
More measures for fatigue prevention	107 (24.3%)	27 (25.5%)	15 (25.0%)	0.97
More measures for nausea prevention	24 (5.5%)	6 (5.7%)	9 (15.0%)	0.02
Better nursing care	69 (15.7%)	12 (11.3%)	11 (18.3%)	0.41
Therapy progress should be reported to patients more often	85 (19.3%)	20 (18.9%)	7 (11.7%)	0.36
More effective therapies	122 (27.7%)	34 (32.1%)	11 (18.3%)	0.16
Doctor consultation with interpreter	9 (2.0%)	26 (24.5%)	1 (1.7%)	< 0.05
Intercultural training for medical staff	47 (10.7%)	24 (22.6%)	11 (18.3%)	0.003
Information in different languages	49 (11.1%)	37 (34.9%)	9 (15.0%)	< 0.05

Forty-five percent of SGM/TGM demanded more time for medical consultations (refer to Table 5). For the majority, the most important source of information was their doctor. A small number of participants contacted a support group. Only 18.6% of the FGM were aware that there was information material available in their preferred language. FGM participants suggested that access to information in different languages was provided to improve cancer care compared to NM and SGM/TGM.

There were no significant differences in the following analysis between patients who participated via an online survey, print-version, and personal interviews. In the subgroup analysis for the type of cancer, no relevant differences between the patients with gynecological and breast cancer were found concerning their satisfaction with the medical consultation (data not shown).

### Tumor etiology

We observed some differences in the explanation model of disease origin between the groups. While NM considered genetic factors as a possible cause (*p* < 0.05) for cancer, PMB stated destiny (*p* < 0.05), stress in the family (*p* < 0.05), and nutrition (*p* = 0.03) as leading factors.

### Side effects, supportive and medical care

We did not find any differences in standard therapies such as surgery, chemotherapy, hormonal, or immunotherapy between the groups. When exploring the participation of PMB in standard medical care, we demonstrated that a similar number of PMB participated in follow-up care examination compared to the NM. A cancer recurrence event occurred just as often between the three groups.

Significant differences were observed in the evaluation of therapy success. Fatigue (*p* = 0.01) and nausea (*p* = 0.03) during the therapy were stated as worse than expected for SGM/TGM compared to NM and FGM. FGM experienced more severe pain than expected during therapy (*p* = 0.03) (refer to Fig. 2b). SGM/TGM experienced nausea and fatigue more often than NM. SGM/TGM asked for therapy options where nausea could be reduced compared to NM. FGM reported cardiac function changes more often and experienced pain more often (refer to Table 3).

**Table 3:** Differences between the groups regarding the participation in the therapy and the experienced side effects

	NM	FGM	SGM/TGM	*p*-value
**Therapy**				
Surgery	406 (92.5%)	94 (92.2%)	55 (91.7%)	0.97
Chemotherapy	368 (84.2%)	87 (82.9%)	51 (85.0%)	0.27
Radiotherapy	130 (30.9%)	33 (33.7%)	15 (25.4%)	0.74
Anti-hormone therapy	98 (23.4%)	27 (26.7%)	17 (29.3%)	0.64
Immunotherapy	171 (40.0%)	28 (29.2%)	25 (41.7%)	0.21
Recurrence	164 (40.3%)	34 (35.1%)	29 (50.0%)	0.24
**Therapy side effects**				
Pain	198 (46.8%)	54 (52.9%)	26 (44.8%)	0.48
Alopecia	288 (68.1%)	72 (70.6%)	42 (72.4%)	0.74
Fatigue	253 (59.8%)	67 (65.7%)	41 (70.7%)	0.19
Blood changes	220 (52.0%)	42 (41.2%)	34 (58.6%)	0.06
Cardiac function changes	16 (3.8%)	9 (8.8%)	1 (1.7%)	0.05
Nausea	158 (37.4%)	50 (49.0%)	31 (53.4%)	0.01
Paresthesia	240 (56.7%)	56 (54.9%)	36 (62.1%)	0.67

### Patient involvement in clinical studies

FGM were considered less often for participation in a clinical study and took part less often in a trial than NM and SGM/TGM (refer to Table 4). A multivariate analysis was performed to examine the hypothesis if migrant background could independently predict the probability of non-participation in a clinical study (refer to Table 5). By including other factors such as language skills and age in a regression model, the migrant background was verified as an independent factor related to less probability of being considered for a clinical trial. FGM were less likely to be offered participation in a clinical trial (OR 1.75, C.I. 0.991–3.80). This effect was most dominant in breast cancer patients (refer to Supplemental Table 2). Another independent factor for not being considered for a trial was poor German skills. In contrast, being SGM/TGM surprisingly increased the chances of being considered for a clinical study, although this trend in a multivariate analysis was not significant. German language skills were an independent factor associated with less probability of a patient being offered to participate in a clinical trial but not associated with less participation.

**Table 4:** Differences between the groups regarding access to clinical trials and cultural specific information

	NM	FGM	SGM/TGM	*p*-value
Offered a clinical trail	216 (50.7%)	29 (28.4%)	34 (56.7%)	< 0.05
Participated in a clinical trail	144 (33.5%)	16 (15.7%)	28 (47.5%)	< 0.05
Offered alternative therapies	162 (39.0%)	37 (37.4%)	27 (48.2%)	0.37
Participated in follow-up care	372 (91.0%)	91 (89.2%)	53 (94.6%)	0.25
Contacted a support group	79 (18.5%)	11 (10.8%)	13 (21.7%)	0.12
Aware of information materials in their preferred language	247 (60.8%)	19 (18.6%)	37 (69.8%)	< 0.05

**Table 5:** Factors predicting the possibility of not being offered or not participating in a clinical trial. The “NM” group is compared to “FGM” and to “SGM/TGM”. Patients with poor German skills were compared to those with fluent German skills. Patients younger than 70 were compared to patients aged 70 and over

	Factors for not being offered participation in a clinical trial				Factors for not participating in a clinical trial			
	OR	C.I. 95% lower	C.I. 95% higher	*p*-value	OR	C.I. 95% lower	C.I. 95% higher	*p*-value
SGM/TGM	0.823	0.474	1.428	0.49	0.582	0.334	1.014	0.06
FGM	1.749	0.991	3.806	0.05	2.172	1.105	4.268	0.02
Poor German language skills	3.265	1.394	7.650	0.006	1.784	0.682	4.666	0.24
Age over 70	1.296	0.863	1.948	0.21	1.305	0.843	2.022	0.23

## Discussion

The number of studies reporting medical needs and disparities of PMB in Germany is insufficient. Based on the experience of previous extensive NOGGO Expression surveys [6, 7], we conducted a new one. This had a special focus on PMB to explore if there are differences in the following: expectations of migrants towards their medical treatment, how they experience communication with their doctors, and how they participate in standard medical care.

We could distinguish between migrants of the first, second, and third-generation with the incorporation of migrant-specific questions which enabled comparisons in these different groups. We encountered some significant differences about FGM in how they experienced the side effects of the therapy and their access to medical information. In comparison to NM and SGM/TGM, FGM struggled more often to overcome language barriers. One major finding was that FGM were generally underrepresented in clinical studies. By performing a subgroup analysis for the type of cancer, we encountered some differences between the patients with gynecological and those with breast cancer. Being a FGM and having breast cancer reduced the chance of being offered participation in a clinical trial. The SGM/TGM patients with gynecological cancer participated more often in clinical studies than those of the FGM group. Some of these differences may be due to the different number of available ongoing clinical studies of different entities during the survey period.

More than one-quarter of the study population had a migrant background. The rate of migrants in this survey is slightly higher than the average number for Germany. This effect is probably due to patient recruitment mostly from the Berlin area, where the migrant percentage is reported to be higher than the average 28% [9]. The FGM were significantly younger, less acculturated, and had poorer German language skills than SGM/TGM.

Most of the patients were very pleased with the quality of medical consultation and their involvement in the decision-making process. We found differences in access to information in a preferred language for FGM, revealing a lack of information due to language barriers.

There were no significant differences between PMB and NM when considering their involvement in standard health care such as follow-up care, chemotherapy treatment, or surgery. A study from Leonhardt et al. [10] reported similar results for overall satisfaction and perception of medical consultation by patients with colorectal cancer. The study group reported that migrants showed less compliance to follow-up care and were less satisfied with some aspects of communication, such as participation in the decision-making process and responses to their questions. For the analysis, the study group considered the FGM and SGM as one. The percentage of migrants in their study was much lower, with 16.3%. The results of our study highlight that there are significant differences between FGM and SGM/TGM. It also demonstrates that different generation migrants should be considered separately.

The data evaluation of 66,953 cancer patients in the study of Mc Grath-Lone L et al. [11] showed that only 30.4% reported having discussions about study participation, and 18.9% took part in the research. Certain groups were under-represented in research populations.

The medical staff was less likely to discuss research participation with older patients. The age of over 70 was not an independent factor for participation in clinical studies in our survey, but poor language skills were.

A German study group [12] evaluated patient knowledge and attitude towards medical research studies among patients with breast cancer and gynecological diseases. They demonstrated that knowledge and willingness to participate strongly depended on age (*p* < 0.001), educational level (*p* < 0.001), and patient group (*p* < 0.001). The level of information that patients received affected their willingness to participate.

Our study gives some arguments to the discussion of why migrants are so under-represented in clinical trials. Based on our study results, we assume that PMB are generally less informed by their doctors regarding possible participation in clinical trials. Despite the high prevalence of other languages in Germany, most informed consents are only in German. We, therefore, suggest that specific training for medical staff and adequate patient information materials may improve the recruitment of migrants in clinical trials.

The present pilot study was designed to gather information about the needs and expectations of PMB and identify possible disparities. We would like to highlight that our study is not a representative survey for the whole and heterogeneous group of migrants in Germany undergoing cancer treatment. Based on our experience from previous published studies we focused on patients with breast and ovarian cancer in order to generate comparable results. Therefore, the results from this survey should not be translated directly to other gynecological malignancies such as cervical or endometrial cancer. Nevertheless, we believe that our results provide relevant information for future projects. In our study, patients were recruited by three different ways of access. We have not observed any differences between these cohorts, but this study was not powered for this purpose. Besides this, we observed a much higher acceptance by migrants and NM when being personally interviewed. Based on our experience, we recommend prioritizing personal interviews for future studies to reduce bias by a high rate of non-compliance and non-participation. Based on our results and according to the GCIG-consensus conference [13] to perform clinical trials in specific migrant populations with gynecological malignancies, we strongly recommend implementing migrant-specific questions in all clinical trials to distinguish patients from different migrant generations.

## Conclusions

Based on our research, this is one of the largest multicentre prospective studies exploring patients expectations with different migrant backgrounds and gynecological malignancies.

The results of our study demonstrated more similarities than differences in the level of satisfaction with medical consultation and the involvement in cancer therapy for both migrants and NM. The physicians were the main source of medical information for the majority of both groups; PMB and NM.

In comparison to NM, PMB were less involved in clinical trials and received less information about studies. Furthermore, PMB stated that they experienced more side effects of cancer therapy than expected. Our results allow the hypothesis that training medical staff in intercultural competence could highly improve supportive care management and quality of life in PMB with cancer and will enable them to have better access to innovative therapies.

## Supplementary Information

**Additional file 1: Supplemental Table 1**. Distribution of study participants according to the way of recruitment. **Supplemental Table 2**. Factors predicting the possibility for not being offered or not participating in a clinical trial.

## Data Availability

The database of the current study is available from the corresponding author on upon request.

## Abbreviations

NOGGO: North-Eastern-German Society of Gynecological Oncology
PMB: Patients with a migrant background
NM: Non-migrants
FGM: First-generation migrants
SGM: Second-generation migrants
TGM: Third-generation migrants
SGM/TGM: Second and third-generation migrants
