# Supplementary material for: Influence of migrant background on patient preference and expectations in breast and gynecological malignancies (NOGGO-expression V study): results of a prospective multicentre study in 606 patients in Germany
Source: BMC Cancer. 2021 Sep 12;21:1018. doi: 10.1186/s12885-021-08731-6 (PMC8436522; doi:10.1186/s12885-021-08731-6)
Supplement: Supplementary file 1 — Additional file 1: Supplemental Table 1. Distribution of study participants according to the way of recruitment. Supplemental Table 2. Factors predicting the possibility for not being offered or not participating in a clinical trial. [file 12885_2021_8731_MOESM1_ESM.docx]

Supplemental Table 1. Distribution of study participants according to the way of recruitment.

|  | Interview | Paper Form | Internet | p-value |
| --- | --- | --- | --- | --- |
| Age (median) | 59 (22-88) | 57 (32-92) | 52 (24-76) | 0.001* |
| NM | 245 (55.7%) | 154 (35.0%) | 41 (9.3%) | 0.46** |
| FGM | 53 (50.0%) | 46 (43.4%) | 7 (6.6%) | 0.46** |
| SGM/TGM | 30 (50.0%) | 23 (38.3%) | 7 (11.6%) | 0.46** |

* Kruskal-Wallis Test, ** Chi-Quadrat Test

Supplemental Table 2. Factors predicting the possibility for not being offered or not participating in a clinical trial

Subgroup analysis for the patients with gynecological cancer (A) and breast cancer (B)

A. Patients with gynecological cancer

|  | **Factors for not being offered participation in a clinical trial** | | | | **Factors for not participating in a clinical trial** | | | |
| --- | --- | --- | --- | --- | --- | --- | --- | --- |
|  | OR | C.I. 95%  lower | C.I. 95 %  higher | p -value | OR | C.I. 95 %  lower | C.I. 95 %  higher | p-value |
| SGM/TGM | 0.554 | 0.253 | 1.217 | 0.14 | 0.377 | 0.178 | 0.798 | 0.01 |
| FGM | 0.992 | 0.462 | 2.127 | 0.98 | 1.695 | 0.728 | 3.944 | 0.22 |
| Poor German language skills | 3.455 | 1.065 | 11.208 | 0.04 | 1.352 | 0.381 | 4.793 | 0.64 |
| Age over 70 | 1.121 | 0.633 | 1.986 | 0.69 | 1.135 | 0.627 | 2.056 | 0.67 |

B. Patients with breast cancer

|  | **Factors for not being offered participation in a clinical trial** | | | | **Factors for not participating in a clinical trial** | | | |
| --- | --- | --- | --- | --- | --- | --- | --- | --- |
|  | OR | C.I. 95%  lower | C.I. 95 %  higher | p -value | OR | C.I. 95 %  lower | C.I. 95 %  higher | p-value |
| SGM/TGM | 1.490 | 0.600 | 3.701 | 0.39 | 1.561 | 0.552 | 4.414 | 0.40 |
| FGM | 4.058 | 1.461 | 11.272 | 0.007 | 3.082 | 0.974 | 9.749 | 0.05 |
| Poor German language skills | 1.854 | 0.472 | 7.283 | 0.37 | 1.742 | 0.369 | 8.233 | 0.48 |
| Age over 70 | 1.550 | 0.840 | 2.859 | 0.16 | 1.396 | 0.718 | 2.718 | 0.33 |
